# A Machine Learning Algorithm Predicting Acute Kidney Injury in Intensive Care Unit Patients (NAVOY Acute Kidney Injury): Proof-of-Concept Study

**DOI:** 10.2196/45979

**Published:** 2023-12-14

**Authors:** Inger Persson, Adam Grünwald, Ludivine Morvan, David Becedas, Martin Arlbrandt

**Affiliations:** 1 Department of Statistics Uppsala University Uppsala Sweden; 2 AlgoDx AB Stockholm Sweden; 3 Department of Anaesthesiology and Intensive Care Södersjukhuset (Stockholm South General Hospital) Stockholm Sweden

**Keywords:** acute kidney injury, AKI, algorithm, early detection, electronic health records, ICU, intensive care unit, machine learning, nephrology, prediction, software as a medical device

## Abstract

**Background:**

Acute kidney injury (AKI) represents a significant global health challenge, leading to increased patient distress and financial health care burdens. The development of AKI in intensive care unit (ICU) settings is linked to prolonged ICU stays, a heightened risk of long-term renal dysfunction, and elevated short- and long-term mortality rates. The current diagnostic approach for AKI is based on late indicators, such as elevated serum creatinine and decreased urine output, which can only detect AKI after renal injury has transpired. There are no treatments to reverse or restore renal function once AKI has developed, other than supportive care. Early prediction of AKI enables proactive management and may improve patient outcomes.

**Objective:**

The primary aim was to develop a machine learning algorithm, NAVOY Acute Kidney Injury, capable of predicting the onset of AKI in ICU patients using data routinely collected in ICU electronic health records. The ultimate goal was to create a clinical decision support tool that empowers ICU clinicians to proactively manage AKI and, consequently, enhance patient outcomes.

**Methods:**

We developed the NAVOY Acute Kidney Injury algorithm using a hybrid ensemble model, which combines the strengths of both a Random Forest (Leo Breiman and Adele Cutler) and an XGBoost model (Tianqi Chen). To ensure the accuracy of predictions, the algorithm used 22 clinical variables for hourly predictions of AKI as defined by the Kidney Disease: Improving Global Outcomes guidelines. Data for algorithm development were sourced from the Massachusetts Institute of Technology Lab for Computational Physiology Medical Information Mart for Intensive Care IV clinical database, focusing on ICU patients aged 18 years or older.

**Results:**

The developed algorithm, NAVOY Acute Kidney Injury, uses 4 hours of input and can, with high accuracy, predict patients with a high risk of developing AKI 12 hours before onset. The prediction performance compares well with previously published prediction algorithms designed to predict AKI onset in accordance with Kidney Disease: Improving Global Outcomes diagnosis criteria, with an impressive area under the receiver operating characteristics curve (AUROC) of 0.91 and an area under the precision-recall curve (AUPRC) of 0.75. The algorithm’s predictive performance was externally validated on an independent hold-out test data set, confirming its ability to predict AKI with exceptional accuracy.

**Conclusions:**

NAVOY Acute Kidney Injury is an important development in the field of critical care medicine. It offers the ability to predict the onset of AKI with high accuracy using only 4 hours of data routinely collected in ICU electronic health records. This early detection capability has the potential to strengthen patient monitoring and management, ultimately leading to improved patient outcomes. Furthermore, NAVOY Acute Kidney Injury has been granted Conformite Europeenne (CE)–marking, marking a significant milestone as the first CE-marked AKI prediction algorithm for commercial use in European ICUs.

## Introduction

Acute kidney injury (AKI) is recognized as a major global public health concern, leading to increased morbidity and mortality, with associated high financial health care costs and a major social impact [1,2]. The incidence of AKI in the intensive care unit (ICU) has increased over the past decade due to increased acuity as well as improved recognition. A multinational epidemiological study has shown that the incidence of AKI in the ICU exceeds 50% (18% in stage 1, 9% in stage 2, and 30% in stage 3) [3]. The development of AKI in ICUs is independently associated with increased ICU length of stay, risk of long-term renal dysfunction (chronic kidney disease and end-stage renal disease), and short- and long-term mortality [4,5].

The definition of AKI has evolved from the risk, injury, failure, loss, and end-stage criteria and the AKI network classification to the Kidney Disease: Improving Global Outcomes (KDIGO) classification [6,7]. These definitions are based exclusively on serum creatinine and urine output.

Timely recognition of AKI has been challenged by limitations associated with the traditional parameters used for diagnosis. Renal impairment typically precedes changes in serum creatinine and urine output. Thus, the current AKI diagnostic and staging strategy only detects AKI after renal injury or impairment has already occurred.

Late AKI diagnosis and its heterogeneous nature have been identified as contributing factors to the limited efficacy observed in drug trials targeting this condition. Studies have indicated that early diagnosis and treatment of reversible AKI reduces mortality [5]. Therefore, an AKI diagnosis based solely on creatinine level and urine volume does not meet the clinical demand. Once AKI has developed, there are no treatments available to reverse or restore renal function other than supportive care, emphasizing the importance of early identification and prevention [8-15].

Extensive research has been carried out to try developing new biomarkers, AKI prediction models, and scoring systems based on risk factors. In recent years, the use of electronic health records (EHRs) has become widespread, and the introduction of artificial intelligence has provided new methods for mining massive medical data and training models based on machine learning algorithms.

AKI is well-suited for prediction and risk forecasting based on routinely collected data contained within ICU EHRs, as the KDIGO consensus definition for AKI allows for temporal anchoring of events.

The Acute Dialysis Quality Initiative convened a group of key opinion leaders and stakeholders to discuss how best to approach AKI research and care in the “big data” era [16]. Acute Dialysis Quality Initiative recommends developing tools for predicting AKI, defined as KDIGO stage 2 or 3, rather than targeting all AKI stages. KDIGO stage 1 can be viewed more as a “risk of AKI.” Traditionally, AKI predictors or risk factors have been more strongly associated with higher-severity AKI [17,18]. This stronger association will likely result in more powerful and robust predictive machine learning algorithms.

Previously published machine learning AKI prediction algorithms have, at least in recent years, shown robust prediction accuracy. However, the absolute majority of the studies are retrospective, single-database studies. Many studies have focused on subspecialized conditions such as cardiac surgery, trauma, and burns. Very few models have been externally or prospectively validated, which limits the generalizability of the models.

To the best of our knowledge, no model has yet taken the final step in the validation process, testing the impact on patient outcomes in randomized clinical trials when used as a clinical decision support tool for making bedside real-time predictions.

In this proof-of-concept study, we have developed, using machine learning methods, an algorithm for early continuous predictions of AKI at KDIGO stage 2 or 3 in a broad critical care setting. This algorithm uses only clinical data routinely collected from the time of admission to the ICU and is designed to be integrated as a clinical decision support tool in EHR systems.

## Methods

### Data Set and Study Population

The algorithm for predicting AKI was developed based on the Massachusetts Institute of Technology Lab for Computational Physiology Medical Information Mart for Intensive Care IV (MIMIC-IV) clinical database [19,20]. This database contains demographics, vital signs, laboratory tests, medications, and more for 53,150 adult ICU patients (76,540 ICU stays) admitted to an ICU or emergency department between 2008 and 2019.

AKI onset was defined as the time of the first onset of KDIGO stage 2 or stage 3 [6,7].

**Figure 1:**
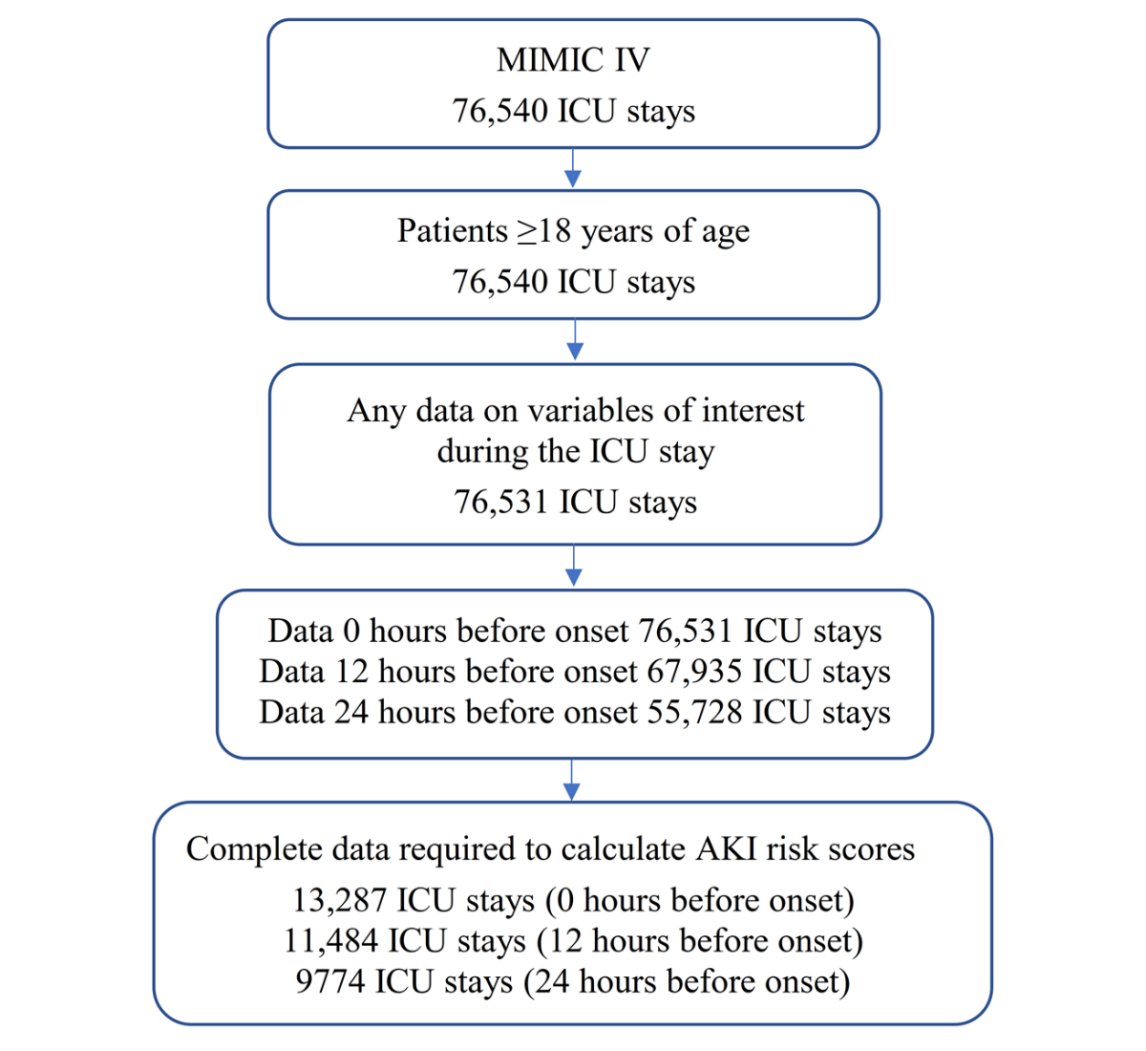
Intensive care unit (ICU) stays included in the analyses. AKI: acute kidney injury; MIMIC IV: Medical Information Mart for Intensive Care IV.

Patients included in the analysis (Figure 1 and Table 1) had at least 1 measurement of each of the variables included in the algorithm and were aged 18 years or older at the time of admission. Differences between the AKI and non-AKI cohorts were assessed by appropriate tests of statistical significance (Welch *t* test for numerical variables, Fisher exact test, or chi-square test for categorical variables). No adjustment was made for multiple comparisons.

To ensure that spurious variables were excluded and the most important variables were included, a preselection of the variables was done in cooperation with medical professionals. Hence, the algorithm was based on the following 22 variables: age, sex, heart rate, respiratory rate, body temperature, systolic blood pressure, diastolic blood pressure, vasopressor use, pH, glucose, lactate, serum creatinine, bilirubin, blood urea nitrogen, leukocytes, thrombocytes, oxygen saturation pulse oximetry, fraction of inspired oxygen, partial pressure of oxygen, International Normalized Ratio, Glasgow Coma Scale, and urine output. Hourly values were used, and a last observation carried forward approach was used for any hours with missing information. For any hours with more than one measurement, hourly averages were used. Feature engineering was performed to obtain 2 additional variables: the creatinine ratio (ratio of the current value of creatinine to the minimum creatinine value during the last 7 days) and the creatinine difference (difference between the current value of creatinine and the minimum creatinine value during the last 2 days). All the variables were then standardized by the mean and SD of the training population. No additional feature engineering was deemed necessary.

**Table 1:** Patient characteristics of population for algorithm development and validation (patients with data 12 hours before onset, n=11,484 intensive care unit [ICU] stays).

Patient characteristics		AKI^a^	Non-AKI	*P* value^b^	
ICU stays, n (%)		3130	8354	—^c^	
**Age (years)**					<.001
	Mean (SD)	63.4 (15.5)	62.0 (16.8)		
	Median (IQR)	64 (54-75)	64 (52-75)		
**Age groups (years), n (%)**					<.001
	18-29	100 (3)	421 (5)		
	30-39	142 (5)	489 (6)		
	40-49	300 (10)	870 (10)		
	50-59	653 (20)	1648 (20)		
	60-69	759 (24)	1911 (23)		
	≥70	1176 (38)	3015 (36)		
**Sex, n (%)**					.02
	Female	1299 (42)	3264 (39)		
	Male	1831 (58)	5090 (61)		
**Length of ICU stay (days)**					<.001
	Mean (SD)	11.48 (10.2)	4.98 (4.6)		
	Median (IQR)	8.4 (4.6-15.0)	3.5 (2.0-6.2)		
**Length of ICU stay (days), n (%)**					<.001
	0-4	880 (28)	5553 (66)		
	5-9	949 (30)	1883 (23)		
	10-14	518 (17)	561 (7)		
	15-19	322 (10)	199 (2)		
	20-24	163 (5)	104 (1)		
	>25	298 (10)	54 (0.6)		
**Time from ICU admission to AKI onset (hours)**					—
	Mean (SD)	105.3 (113.9)	—		
	Median (IQR)	63.8 (34.5-135.0)	—		
**Comorbidities^d^, n (%)**					<.001
	Chronic obstructive pulmonary disease	706 (22.6)	1805 (21.6)		
	Chronic kidney disease	609 (19.5)	1278 (15.3)		
	Diabetes mellitus	739 (23.6)	1831 (21.9)		
	Cerebrovascular disease	374 (11.9)	1096 (13.1)		
	Ischemic heart disease	822 (26.3)	2227 (26.7)		
	Hypertension	1457 (46.5)	3887 (46.5)		
	Chronic liver disease	395 (12.6)	826 (9.9)		
	Major cancers	591 (18.9)	1537 (18.4)		
	Peripheral vascular disease	471 (15)	1270 (15.2)		
	Heart failure	798 (25.5)	1881 (22.5)		
	Sepsis	825 (26.4)	1566 (18.7)		
**Admission to type of ICU, n (%)**					<.001
	Cardiac vascular ICU	328 (10.5)	1517 (18.2)		
	Coronary care unit	380 (12.1)	815 (9.8)		
	Medical ICU	1029 (32.9)	2154 (25.8)		
	Medical or surgical ICU	552 (17.6)	1411 (16.9)		
	Neuro ICU	151 (4.8)	285 (3.4)		
	Surgical ICU	407 (13)	1192 (14.3)		
	Trauma surgical ICU	283 (9)	980 (11.7)		
**Death during hospital stay, n (%)**					<.001
	Yes	1233 (39.4)	1836 (22)		
	No	1897 (60.6)	6518 (78)		

^a^AKI: acute kidney injury.

^b^Differences between the AKI and non-AKI cohorts, as assessed by Welch *t* test for numerical variables or Fisher exact test or chi-square test for categorical variables.

^c^Not applicable.

^d^Comorbidities are defined by International Statistical Classification of Diseases, ninth revision codes registered during the ICU stay.

### Machine Learning Algorithm Development

The algorithm was developed using a hybrid ensemble model [21,22] consisting of a Random Forest (Leo Breiman and Adele Cutler) and an XGBoost model (Tianqi Chen) [23]. This method effectively combines both models, and the final risk score is a weighted combination of the predictions from both models. This method was chosen based on its strong performance with tabular data. Each of the 2 models could face difficulties predicting in specific situations, and their combination acts as a safety net to mitigate the mistakes of each other, reducing the impact of their potential individual errors. Data were preprocessed using R (The R Project), and the models were executed using XGBoost [23] and Sci-Kit Learn (David Cournapeau) [24] backends in Python (version 3.8; Python Software Foundation).

The model’s hyperparameters were selected using a sparse grid search, exploring a reasonable number of hyperparameter combinations while excluding combinations that would obviously underperform or not substantially enhance performance. The XGBoost model used the following nondefault hyperparameters: “max_depth” = 8, “learning_rate” = 0.2, “reg_lambda” = 1.2, and “min_child_weight” = 4. Training stopped if the validation error had not decreased for the last 10 training rounds. Area under the receiver operating characteristic curve (AUROC) was used as the evaluation metric. The Random Forest, executed with Sci-Kit Learn, used the following hyperparameters: “max_features” = 0.5, “min_samples_leaf” = 10, and “n_estimators” = 300. The models were then combined with weights of 0.25 for the Random Forest model and 0.75 for the XGBoost model.

The data were split into 3 separate data sets: 1 training set to train the model, 1 validation set to continuously evaluate performance for different hyperparameter combinations, and 1 test set, which was held out to test the final model’s performance. Random onset matching [25] was used, randomly selecting 4‑hour sequences with the last time point 12 hours before AKI onset for patients with AKI or at any point during the entire ICU stay for patients without AKI. The time points were sampled to maintain a similar distribution of time since admission to the ICU in both populations. Since the algorithm was initially planned for implementation in the Nordic countries, data were sampled to maintain a prevalence of AKI of 22% in all 3 data sets, resembling the prevalence of AKI stages 2 and 3 in Nordic ICU patients [26]. This also facilitated comparisons between the data sets, as the AUROC, area under the precision-recall curve (AUPRC), and accuracy are influenced by prevalence. A prediction horizon of 12 hours was chosen to predict AKI as early as possible as well as to minimize performance degradation observed in longer prediction horizons. The training data consisted of 9996 sequences (n=9996 ICU stays) of 4-hour data (AKI, n=2199 sequences and non-AKI, n=7797 sequences). The validation data consisted of 2128 sequences (n=2128 ICU stays) of 4-hour data (AKI, n=468 sequences and non-AKI, n=1660 sequences). The test data consisted of 2105 sequences (n=2105 ICU stays) of 4-hour data (AKI, n=463 sequences and non-AKI, n=1642 sequences) and were only used in the final evaluation of the chosen model.

### Performance

To assess performance, receiver operating characteristic (ROC) were calculated, that is, the proportion of true positives (sensitivity) in relation to the proportion of false positives (1– specificity). Based on the ROC curve, an operating point (threshold) was chosen for classifying patients with a high risk of developing AKI. True positives were patients with AKI that were accurately predicted by the algorithm 12 hours before AKI onset, and false positives were patients without AKI that were wrongly predicted by the algorithm to be at risk of developing AKI. The operating point for the algorithm was chosen to keep sensitivity (the proportion of true positives) around 0.80 while maximizing specificity (the proportion of true negatives) to minimize the false alert rate while ensuring high sensitivity. The algorithm should ideally provide a high proportion of true positives and a low proportion of false positives, corresponding to a large AUROC. The AUPRC is also important, where a large area represents both high recall (low false negative rate) and high precision (low false positive rate). High scores for both recall and precision show that the algorithm yields accurate results (high precision) and captures the majority of all positive results (high recall). Accuracy is the proportion of correct predictions, and positive predictive value is the proportion of predicted AKI cases that are true AKI cases.

### Variable Importance

**Figure 2:**
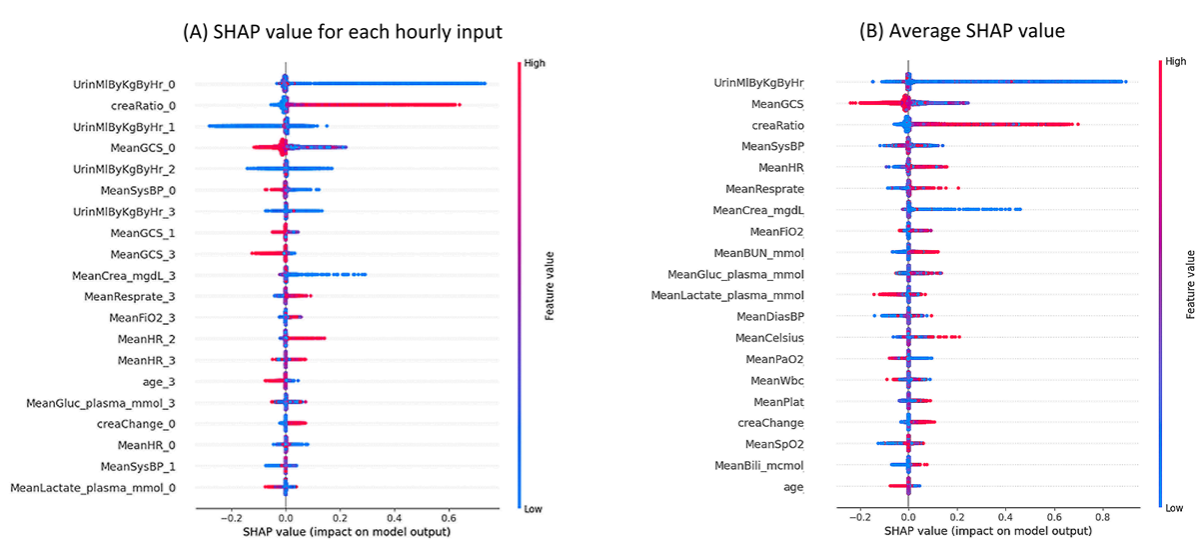
Shapley Additive exPlanations (SHAP) values for the hold-out data. Each point on the graph corresponds to the SHAP value for a specific variable and prediction. The red color indicates a high variable value, while blue indicates a low value. A high absolute SHAP value signifies a variable’s high importance. A positive SHAP value increases the predicted risk, while a negative SHAP value decreases it. (A) SHAP values produced with input values of each variable from all 4 time points (with t being the last hour of the 4-hour period). (B) SHAP values averaged over the 4-hour period. Example of interpretation: urine output at *t* (12 hours before acute kidney injury onset) is the most important parameter, as it has the largest absolute SHAP value. The blue color indicates that a low urine output value will increase the predicted risk. Creatinine ratio 12 hours before onset is the second most important parameter, as it has the second largest absolute SHAP value. The red color indicates that a high creatinine ratio value will increase the predicted risk.

For the sake of model interpretability, variable importance was calculated using the kernel SHAP (Shapley Additive exPlanations) method [27]. The SHAP method calculates Shapley values for each prediction, and the Kernel SHAP uses a weighted linear regression to compute these values. Figure 2 presents an example of a graphic obtained with the SHAP values calculated on the hold-out data. Figure 2A illustrates the distribution of the SHAP values for each variable. To evaluate the global contribution of each variable independently of time, the SHAP value was summed over time for each variable, yielding Figure 2B. According to Figure 2, urine output, creatinine ratio, and Glasgow Coma Scale are the most contributing variables to the model for the hold-out data set.

### Ethical Considerations

As this study is based on a publicly available database, an ethics review was not sought. The MIMIC-IV contains deidentified data, where patient identifiers have been carefully eliminated in compliance with the HIPAA (Health Insurance Portability and Accountability Act) safe harbor provision. The process of gathering patient data and establishing the research database underwent evaluation by the institutional review board at the Beth Israel Deaconess Medical Center. They granted an exemption from the requirement for informed consent and gave their approval for the data sharing endeavor [19,20].

## Results

**Figure 3:**
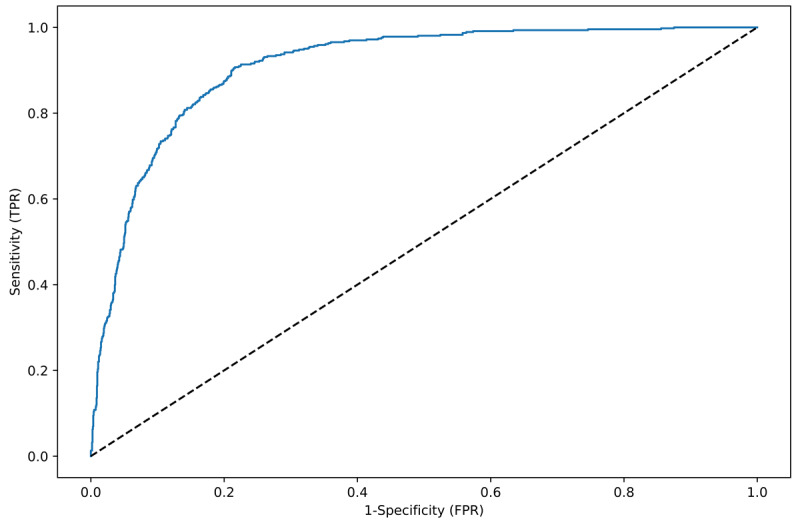
Receiver operating characteristic (ROC) curve for algorithm predicting acute kidney injury (AKI) and hold-out test data predicting AKI 12 hours before onset. AUROC: area under the ROC curve; FPR: false positive rate; TPR: true positive rate.

The AUROC for the developed algorithm was as high as 0.91 (Figure 3 and Table 2) when predicting 12 hours before onset. The AUPRC was 0.75 on training data and 0.71 on test data when predicting 12 hours before onset (Table 2). The sensitivity, specificity, and accuracy of the algorithm were all high (sensitivity 0.84-0.85, specificity 0.85-0.87, and accuracy 0.84-0.85; Table 2). The chosen operating point yielded a positive predictive value of 0.61 on training data and 0.63 on test data when predicting 12 hours before onset (Table 2). This metric was expected to be lower than the sensitivity, specificity, and accuracy due to the class imbalance. A sensitivity of 80% (Table 2) results in 20% false positives, and since most patients were negative cases (non-AKI), there would be an overproduction of predicted AKI cases. Comparing the distribution of AKI predictions made by the algorithm with the distribution of actual AKI cases (prevalence), we can see that the algorithm predicted 29% of AKI cases in training data and 28% in test data (Table 2), which is somewhat larger than the prevalence of 22%.

**Table 2:** Validation performance for algorithm predictions 12 hours in advance.

Performance metric	Training data	Hold-out test data
AUROC^a^ curve	0.91	0.91
AUPRC^b^	0.75	0.71
Accuracy (95% CI)^c^	0.84 (0.83-0.86)	0.85 (0.84-0.87)
Sensitivity^c^	0.80	0.79
Specificity^c^	0.85	0.87
PPV^c,d^	0.61	0.63
Proportion predicted AKI^e^ cases	0.29	0.28

^a^AUROC: area under the receiver operating characteristic.

^b^AUPRC: area under the precision-recall curve.

^c^Operating points for the algorithm are chosen to keep sensitivity around 0.80.

^d^PPV: positive predictive value.

^e^AKI: acute kidney injury.

## Discussion

### Principal Results

In this study, we developed a machine learning algorithm, NAVOY Acute Kidney Injury, for early continuous predictions of stage 2 and 3 AKI in ICU patients. The algorithm was trained on data from a broad critical care setting (the MIMIC-IV clinical database) and was designed for integration as a clinical decision support tool within EHR systems in ICUs. To optimize its use as a prospective clinical decision support tool, it was designed to make fully automated continuous predictions based on real-time data routinely collected in ICU EHR systems, using variables collected from time of admission and 4 hours of input. This allows for high-performance risk assessments for AKI in adult patients to be provided to clinical staff within only a few hours after ICU admission. Specificity (proportion of true negatives) was prioritized to reduce false alarms, which is especially relevant in clinical decision support tools since interventions might carry some risk. This also decreases the risk of alarm fatigue, which is a well-known phenomenon in critical care settings.

The AUROC of NAVOY Acute Kidney Injury was 0.91 for predictions 12 hours before AKI onset, and this result was consistent between training and test data, indicating that the algorithm yields a high proportion of true positives and a low proportion of false positives. NAVOY Acute Kidney Injury has been externally validated at Skåne University Hospital in Sweden (ClinicalTrials.gov NCT05424874, data on file) and obtained Conformite Europeenne (CE)–marking, making it the first CE-marked AKI prediction algorithm for commercial use in European ICUs.

### Limitations

NAVOY Acute Kidney Injury was trained on a US adult population (MIMIC-IV), and the evaluation was performed on a hold-out data set from the same population, which may limit its generalizability and suggest a need for additional external validation (ongoing research).

Additionally, the evaluation was based on retrospective data, which could lead to inconsistencies in data recording and necessitate prospective validation before putting the algorithm to use in clinical practice. Furthermore, the calculation of the creatinine ratio used the first creatinine value following ICU admission as the baseline, not the first value in the patient’s hospital stay, potentially missing some cases on the first day of their ICU stay.

### Comparison With Previous Work

Most previously published machine learning AKI prediction algorithm studies are retrospective and single-database studies, often focusing on specific conditions such as cardiac surgery, trauma, and burns. Few models have been externally or prospectively validated, limiting their generalizability.

In a review of 19 published machine learning AKI prediction algorithms by Gameiro et al [28], one model was prospectively validated in an ICU setting [29]. This model was developed to predict AKI based solely on creatinine. Baseline creatinine values were defined as the lowest creatinine value identified in the 3 months before, not including admission. Predictions were made upon ICU admission (AUROC 0.80), on the first morning in the ICU (AUROC 0.94), and after 24 hours of ICU stay (AUROC 0.95).

Yu et al [30] recently published a review of machine learning models for AKI. A total of 13 algorithms were studied in a critical care setting comparable to our patient cohort. Performance was reported as AUROC, ranging from 0.69 to 0.926. The model with the highest reported AUROC was designed to predict whether patients with AKI stages 1 or 2 will progress to AKI stage 3 [31]. One model, designed to make daily predictions, was externally and prospectively validated, with an AUROC of 0.86 [32].

As pointed out by Moor et al [25], it can be difficult to compare studies based on measures such as AUROC or accuracy, as these measures are directly affected by the prevalence of the studied condition. Even studies from the same database can be difficult to compare due to differences in data extraction and data preprocessing methods. In situations where there is an imbalance, such as in AKI prediction, where the number of patients without AKI is substantially greater than those with AKI, the AUPRC should be reported. While AUROC is primarily affected by specificity and sensitivity, AUPRC is more dependent on the balance between precision and recall. An algorithm can have a very high AUROC, but a much lower AUPRC if the prevalence is very low. However, the NAVOY Acute Kidney Injury algorithm has a high AUROC as well as a high AUPRC, indicating that the algorithm provides accurate results (high precision) and returns a majority of all positive results (high recall). Direct comparisons with previously published AKI algorithms are, however, challenging since none of them have presented AUPRC values.

To the best of our knowledge, no machine learning AKI prediction algorithm has yet taken the final step in the validation process, testing the impact on patient outcomes in randomized clinical trials when used as a clinical decision support tool for making real-time bedside predictions. Dascena Inc had planned a clinical trial for the Previse AKI prediction algorithm [33] but this study has been withdrawn (ClinicalTrials.gov NCT04200950). A clinical trial has been conducted with the Mayo Clinic AKI Sniffer [34,35], but results have not yet been published (ClinicalTrials.gov NCT01621152).

### Future Work

While NAVOY Acute Kidney Injury shows promise, further research is needed to assess its generalizability and clinical utility. External validation in diverse patient cohorts and prospective clinical trials are essential steps toward establishing the algorithm as a reliable clinical decision-support tool. In future implementations of the algorithm at different institutions, an initial “silent” period is planned, during which the predictions will not be presented. This period will facilitate a prospective comparison between the predictions and the actual onset of AKI and will thereby enable calibration of the model to ensure that the algorithm functions as expected at each institution before going live. We have developed a technical platform for real-time predictions, which is currently being tested with our sepsis prediction algorithm, NAVOY Sepsis [36], in the ICU at the Southern General Hospital in Sweden (ClinicalTrials.gov NCT05095220). In future research, we intend to clinically validate NAVOY Acute Kidney Injury in a similar fashion. The integration of NAVOY Acute Kidney Injury into ICU settings holds potential for improving real-time patient care and outcomes.

### Conclusions

AKI affects a large proportion of ICU patients and is associated with significant morbidity and mortality. Currently, AKI is diagnosed using the KDIGO classification based on serum creatinine and urine output, parameters that typically lag behind renal injury. We have developed a machine learning AKI prediction algorithm, NAVOY Acute Kidney Injury, that predicts the risk of AKI (KDIGO stage 2 or stage 3) with high accuracy up to 12 hours before onset. The algorithm uses variables routinely collected and contained in ICU EHRs and could serve as a valuable tool for strengthened patient monitoring, earlier detection, and intervention, potentially improving patient outcomes. NAVOY Acute Kidney Injury is the first CE-marked AKI prediction algorithm for European ICUs, but further validation and prospective studies are necessary to confirm its generalizability and clinical utility.

## Abbreviations

AKI: acute kidney injury
AUPRC: area under the precision-recall curve
AUROC: area under the receiver operating characteristics
CE: Conformite Europeenne
EHR: electronic health record
HIPAA: Health Insurance Portability and Accountability Act
ICU: intensive care unit
KDIGO: Kidney Disease: Improving Global Outcomes
MIMIC-IV: Medical Information Mart for Intensive Care IV
ROC: receiver operating characteristic
SHAP: SHapley Additive exPlanations

## Data Availability

The data sets analyzed during this study are available through PhysioNet [20].
